# New Insights into Diffuse Large B-Cell Lymphoma Pathobiology

**DOI:** 10.3390/cancers12071869

**Published:** 2020-07-11

**Authors:** Antonio Giovanni Solimando, Tiziana Annese, Roberto Tamma, Giuseppe Ingravallo, Eugenio Maiorano, Angelo Vacca, Giorgina Specchia, Domenico Ribatti

**Affiliations:** 1 Department of Biomedical Sciences and Human Oncology, Section of Internal Medicine ‘G. Baccelli’, University of Bari Medical School, 70124 Bari, Italy; angelo.vacca@uniba.it; 2 Istituto di Ricovero e Cura a Carattere Scientifico-IRCCS Istituto Tumori “Giovanni Paolo II” of Bari, 70124 Bari, Italy; 3 Department of Basic Medical Sciences, Neurosciences, and Sensory Organs, University of Bari Medical School, 70124 Bari, Italy; tiziana.annese@uniba.it (T.A.); roberto.tamma@uniba.it (R.T.); 4 Department of Emergency and Transplantation, Pathology Section, University of Bari Medical School, 70100 Bari, Italy; giuseppe.ingravallo@uniba.it (G.I.); eugenio.maiorano@uniba.it (E.M.); 5 Department of Emergency and Transplantation, Hematology Section, University of Bari Medical School, 70100 Bari, Italy; giorgina.specchia@uniba.it

**Keywords:** DLBCL, tumor microenvironment, angiogenesis, cell adhesion mediated drug resistance, tumor progression

## Abstract

Diffuse large B-cell lymphoma (DLBCL) is the most common non-Hodgkin lymphoma (NHL), accounting for about 40% of all cases of NHL. Analysis of the tumor microenvironment is an important aspect of the assessment of the progression of DLBCL. In this review article, we analyzed the role of different cellular components of the tumor microenvironment, including mast cells, macrophages, and lymphocytes, in the tumor progression of DLBCL. We examined several approaches to confront the available pieces of evidence, whereby three key points emerged. DLBCL is a disease of malignant B cells spreading and accumulating both at nodal and at extranodal sites. In patients with both nodal and extranodal lesions, the subsequent induction of a cancer-friendly environment appears pivotal. The DLBCL cell interaction with mature stromal cells and vessels confers tumor protection and inhibition of immune response while delivering nutrients and oxygen supply. Single cells may also reside and survive in protected niches in the nodal and extranodal sites as a source for residual disease and relapse. This review aims to molecularly and functionally recapitulate the DLBCL–milieu crosstalk, to relate niche and pathological angiogenic constitution and interaction factors to DLBCL progression.

## 1. Introduction

Diffuse large B-cell lymphoma (DLBCL) classified by the 2008 World Health Organization (WHO) classification as one of the B-cell lymphomas types is the most common non-Hodgkin B-cell lymphoma (NHL), accounting for about 40% of all cases of NHL [1]. DLBCL characteristically presents with advanced stage, both in nodal and in extranodal symptomatic disease, with a median age of 60, representing an important disease holding a practical objective of treatment represented by a curative approach, while minimizing the toxicity profile [2]. Most DLBCLs arise from germinal B cells at different stages of differentiation where recurrent genetic alterations contribute to the molecular pathogenesis of the disease [3,4]. The gene expression profiling technique allowed identifying at least two molecular subtypes of DLBCL with different prognoses [5]. The first is the lymphoma derived from normal germinal center B cells (GCB) and the second one is the lymphoma derived from activated B cells (ABCs) that arise from post-germinal center B cells that are blocked during plasmocytic differentiation. The two subtypes have different oncogenic mechanisms [6].

Specific markers, including CD10, LM02, and BCL6 are expressed in GCB patients who have a better response to conventional chemotherapy, whereas ABC patients express lower levels of BCL6 and are refractory to chemotherapy [5,7]. The ABC type showed constitutive activation of NF-κB which may be related to the presence of mutations of multiple genes regulating this pathway [8,9]. Constitutively activated STAT3 is correlated with a more advanced clinical stage and overall poor survival in DLBCL [10,11]. In ABC DLBCL, the activation of the Janus kinases (JAKs)/STAT3 pathway correlates with autocrine production of intereukin-6 (IL-6) and IL-10, which promotes cancer progression [12,13]. The STAT3 gene is a transcriptional target of BCL6 and is highly expressed and activated in ABC DLBCL and BCL6-negative normal germinal center B cells [12]. Moreover, STAT3 is strongly linked to tumor angiogenesis and metastasis and is related to poor prognosis in different tumors [14,15]. Activation of STAT3 contributes to hypoxia-inducible factor 1 alpha (HIF-1α) and vascular endothelial growth factor (VEGF) expression in tumor cells, while VEGF in turn activates STAT3 in endothelial cells. Finally, STAT3 inhibits the expression of the anti-angiogenesis transcription factor p53 [16].

In DLBCL, the gene expression signatures “stromal 1” and “stromal 2”, related to extracellular matrix and angiogenesis-related genes, respectively, were identified [17]. Fibrosis and myelo-histiocytic infiltration, representing the “stromal 1” signature, correlated with a positive clinical outcome [18], while the “stromal 2” signature, characterized by increased vasculogenic activity, correlated with dismal prognosis in subjects treated with the R-CHOP (rituximab, cyclophosphamide, doxorubicin, vincristine, and prednisone) protocol [17]. Monocytic myeloid-derived suppressor cells and tumor-associated macrophages (TAMs) play a crucial role in the “stromal 2” signature [19,20]. Overall, as in other hematological niche addicted malignancies [21,22,23,24], the current evidence pinpoints that DLBCL disease progression is a multistep transformation process characterized by a complex vicious cycle between lymphoma cells and the tumor milieu.

Here, we show the latest findings on the disease evolution of DLBCL, by providing a specific focus on the role of new players within the cancer immune microenvironment in order to envision novel theragnostic windows.

## 2. Bridging the Gaps between Disease Biology and Clinical Translation: New and Old Tricks in DLBCL Classification

A correct diagnosis of DLBCL requires, in addition to the availability of qualitatively and quantitatively adequate tissue, a correct application of the most recent classification principles provided by the use of any ancillary diagnostic techniques. In particular, modern histopathological diagnostics of lymphomas requires knowledge and combination of morphological, phenotypic molecular, cytogenetic, and clinical profiling. This methodological approach constitutes the founding principle of the World Health Organization (WHO) and was translated into the “blue book” “WHO Classification of Tumors of the Hematopoietic and Lymphoid Tissues” [1]. Recent progresses in understanding the immunogenetic mechanisms and genetic molecular alterations of hematopoietic and in particular lymphoid neoplasms allowed a pathogenetic approach to the DLBCL taxonomy. Many lymphomas are considered distinct entities, characterized by immunophenotypic profiles and known genetic alterations, identifiable with laboratory techniques now widely used, with good reproducibility. DLBCL parallels the complex NHL biological architecture, being differentiated into the GCB type and the ABC/non-GC type, by means of an immunohistochemical algorithm, which is a distinction that can influence the therapeutic choice [1,25,26]. Furthermore, the co-expression of MYC and BCL2 identifies a new prognostic “subset” (“double-expressor” lymphomas) [27]. Although the understanding of the mutation scenario was also widened and deepened, the translational relevance in the clinical subset still represents an unmet medical need.

Recently, NGS studies uncovered different profiles of genomic alterations to be relevant in both the GCB and the non-GCB/ABC subtypes [28]. Alteration in histone-lysine *N*-methyltransferase (EZH2), as well as the translocation of BCL2 and GNA13 mutation, is a fundamental molecular fingerprint described in GCB. Conversely, MYD88, CD79a, CARD11, and TNFAIPA3 mutations play a pivotal role in non-GCB/ACB by activating the BCR and NF-κB pathways [25]. The importance of a subdivision of diffuse large B-cell lymphomas, NOS in the two groups (GCB and non-GCB/ABC), is confirmed. This distinction, with possible therapeutic consequences, can be obtained in routine diagnostics by applying an immunohistochemical algorithm based on a relatively simple and reliable antibody panel (CD10, BCL6, and IRF4/MUM1) [28]. Moreover, among the DLBCL NOS, the immunohistochemical co-expression of MYC and BCL2, deemed biologically and clinically relevant, identifies the category of double-expressor disease, harboring an unfavorable prognostic impact [27].

### 2.1. Molecular Pathogenesis: Novel Insights

Double/triple-hit high-grade B-cell lymphoma (HGBL-DH/TH) constitutes approximately 8% of DLBCL, harboring MYC, BCL2, and/or BCL6 translocations. Most of them belong to the GCB molecular subgroup and, clinically, despite the generally superior prognosis of GCB DLBCLs, patients with HGBL-DH/TH have a poor outcome [29]. Double-hit lymphomas show a distinct gene expression profile when dissected by RNA-seq. For example, 157 de novo GCB DLBCLs, including 25 HGBL-DH/TH BCL2, were analyzed to define gene expression differences between HGBL-DH/TH BCL2 and other GCB DLBCLs [30]. When RNA-seq was applied to RNA extracted from fresh frozen biopsy samples, 104 genes that were most significantly differentially expressed between HGBL-DH/TH BCL2 and other GCB DLBCLs were identified [30]. Double-hit gene signature-positive (DHITsig-pos) DLBCLs are characterized by a peculiar cell of origin and a distinct mutational landscape, after genetic feature association with DHITsig status. DHITsig-pos tumors were universally positive for CD10 staining, and the majority were MUM1 (IRF4)-negative. CD10+/MUM1− cases were significantly more frequent in DHITsig-pos tumors. Genes associated with the GC intermediate zone had higher expression within the DHITsis-pos tumors. These findings demonstrate that DHITsig-pos tumors are B cells transitioning from the GC dark zone to the GC light zone. Along with the expected enrichment of mutation in MYC and BCL2, mutations of genes involved in chromatin modification (e.g., CREBBP, EZH2, DDX3X, TP53, and KMT2D) were more frequently harbored by DHITsig-pos tumors [30,31,32]. Specifically, missense mutations in EZH2, DEAD-box helicase 3 X-linked (DDX3X), and lysine methyltransferase 2D (KMT2D), as well as both missense and truncating mutations in CREB-binding protein (CREBBP) and TP53, point toward different clinical features of the corresponding DLBCL subjects [30]. Moreover, DHITsig identified a group of DLBCL with peculiar clinical features. The variable molecular signatures, which identify HGBL and are constituted by the karyotype, the immunohistochemistry, and the DHIT signature [30], uncovered novel clinical scenarios to be driven by a still evolving genomic landscape in DLBCL, and they enable a rational patient management, based on consolidated [26,33] and novel therapeutic approaches [25,34].

In the frame of this thinking, regulation of chromatin status plays a pivotal role in the correct development and differentiation of mature B cells, and it is extensively investigated with therapeutic purposes. In B-cell tumors, a plethora of mutations affect genes involved in chromatin regulation and in normal B-cell development [31,32,33]. Specifically, EP300 and CREBP are main acetylation regulators and, therefore, modulate gene expression, as well as histone methylators such as KMT2D, SUZ12, and EZH2 [35,36,37]. These genes are mutated in 25–30% of DLBCL cases. Notably, CREBBP and EP300 positively modulate multiple biological programs in the germinal center, through acetylation of histone and nonhistone proteins. Moreover, CREBBP and EP300 mutations contribute to lymphomagenesis by perturbing the expression of genes that are relevant to normal biology (i.e., BCL6 and p53). Inactivation of CREBBP and EP300 rarely coexists in human DLBCL, suggesting that cells require a certain amount of acetyltransferase activity [38]. Remarkably, GC B cells essentially require a minimum amount of acetyltransferase activity [39] and CREBBP-mutated B cells are addicted to the residual activity of EP300, envisioning potential therapeutic windows driven by CREBBP-mutated GC B cells on EP300 [39]. Thus, double KO of CREBBP and EP300 is required to abrogate GC formation detected by BCL6 immune staining. Furthermore, CREBBP-deficient cells are preferentially sensitive to inhibitors targeting HAT/BRD domains of CREBBP/EP300 [39]. In DLBCL with CREBBP genetic inactivation by mutation, pharmacologic inactivation of EP300 may lead to lymphoma cell death. Additionally, EP300 polymorphism was uncovered to decrease the balance between acetylation and deacetylation in the tumor niche, impacting disease progression [40]. Epigenetic dysregulation can, therefore, represent one of the driver lesions in high-risk DLBCL, and the restoration of physiological chromatin remodeling is an attractive target for novel therapy.

In tumor patients, based on evidence from other solid [41,42,43] and hematological malignancies [44,45,46,47], cell-free DNA (cfDNA) and extracellular vesicles are released by tumor apoptotic cells; DLBCL makes no exception [48,49]. Circulating tumor DNA (ctDNA) is distinguished from other cfDNA by the presence of somatic mutations representative of tumor biology absent in normal cells [50]. Liquid biopsy was employed as a new tool for genotyping and evaluating minimal residual disease in DLBCL [51,52]. Kurtz et al. uncovered ctDNA^high^ DLBCL to be characterized by a prognostically unfavorable outcome [52]. Remarkably, the non-tumor cfDNA might additionally originate from the neoplastic site, expanding the concept of liquid biopsy to the microenvironment compartment [53]. Liquid biopsy and molecular deconvolution [51], dissecting the genomic architecture of hematological malignancies, are becoming tools able to predict the prognosis [7,54,55].

### 2.2. Molecular Prognostic Models

Efficient clinical prognostic tools were uncovered to be relevant in driving patient management. The international literature highlighted some molecular characteristics of DLBCLs that condition their prognosis and, in perspective, the therapy [1]. Adequate histological diagnosis must include in the report an evaluation of the parameters useful to guide the therapeutic choice in order to confirm the cell of origin, its immunophenotype, the presence of double expressors, and the proliferation index, as well as sometimes specific FISH characteristics addressed by BCL2, BCL6, MYC, and IG-heavy/kappa/lambda (IGH/IGK/IGL) DNA probes [1,56]. The prognostic impact of the biological characteristics holds relevant translational consequences. To this end, a proper stratification included specific characteristics of investigation on the cancer cells that were uncovered to be CD20- and/or CD79a-expressing B lymphocytes [26,57]; additionally, anti-CD5 was deemed important when expressed, thus allowing the identification of a clinically more aggressive CD5+ DLBCL subset [57]. Moreover, while characterizing the cell of origin phenotype, CD10, BCL6, and MUM1 play a pivotal role, by driving the GC-type identification, differentiated by CD10 and/or BCL6 expression in >30% of DLBCL cells, while their low expression, along with >30% expression of MUM1 documentation, indicates a non-GC-type [58].

MYC/BCL2 evaluation in DLBCL using immunohistochemical staining was employed to exactly define double expression and to identify subgroups with dismal prognosis, often belonging to the non-GC-type subgroup [59]. A percentage of cells with intense MYC positivity >70% is often associated with translocation [60].

The percentage of Ki67-positive tumor cells (clone MIB1) should also be considered. In the event of uneven distribution in the tissue, it is advisable to report a percentage value representative of the average, while signaling the uneven distribution of the positivity signal [61].

Several alternative prognostic models already exist for DLBCL. A new one was uncovered to be significant, showing, in 199 cases, the relevance of the immunohistochemistry according to the Hans algorithm and MYC/BCL2 evaluation. The cell of origin evaluated by Nanostring, FISH analysis assessing BCL2, BCL6, and c-MYC, and the targeted sequencing from a custom platform based on univariate analysis identifying gene mutations significantly correlated to poor or favorable prognosis [62]. According to that stratification system, the authors elaborated an m3D-IPI uncovering sex, age, extranodal sites, LDH, advanced stage, double hit, and mutation in KMT2D, PIM1, and MEF2B as being significantly related to high-risk disease in R-CHOP-treated patients. Despite statistically powered validation studies being required, this novel approach performed better than traditional IPI in this patient cohort (C-index 0.87 vs. 0.77, respectively). The increasing number of biological acquisitions, combined with clinical characteristics of patients, will allow a better treatment tailoring.

Recently, since limited data are available on comprehensive genetic signatures, Chapuy et al. proposed a novel molecular gene signature deconvoluting the DLBCL heterogeneity. While dissecting the complex genomic architecture, these authors uncovered an integrated approach combining analyses of recurrent mutations, somatic copy number alterations (SCNAs), and structural variants (SVs) to efficiently reveal DLBCL taxonomy, and they highlighted five genetically distinctive clusters (C1–C5) [63]. Specifically, these genetically distinct DLBCL subsets predict different outcomes, provide novel insights into lymphomagenesis, and suggest certain combinations of targeted therapies [63,64]. In more detail, among ABC DLBCLs, the C1 subtype DLBCL was deemed to be associated with favorable prognosis and was characterized by MYD^non-L265P^, NOTCH2, and SPEN mutations, as well as BCL6 SVs, and this phenotype might origin from marginal-zone lymphoma and from an ancestor of extrafollicular origin [63]. Conversely, C5 subtype DLBCLs correlated with unfavorable clinical outcome, harboring BCL2^gain^, MYD88^L265^, CD79B^mut^, and TBL1XR1^mut^, and they were associated with extranodal tropism and genes overexpressed in the BCL2-overexpressing group [65,66]. Contrariwise, within the GCB DLBCLs, the C4 subtype was associated with more favorable PFS, and it was characterized by mutations in NF-κB, JAK/STAT, and RAS pathway components and histone genes. The C3 subgroup paralleled the C5 dismal prognosis, being associated with BCL2 SV and mutations, PTEN CN loss and mutation, and chromatin-modifying enzyme alterations. Lastly, Chapuy et al. also identified a remarkable feature from a C2 subtype with a distinct clinical trajectory, being composed by bi-allelic TP53 inactivation, 9p21.23/CDNKN2A copy loss, and increased genomic instability reflected by recurrent SCNAs and frequent genome doublings [63]. Next, to validate the genetic substrate in an independent dataset and develop a robust molecular classifier allowing prediction in new samples, Chapuy et al. also genetically confirmed identity-associated marker genes and biology of the C1–C5 DLBCL clusters in a combined larger cohort [32,67]. This independent analysis sanctioned a parsimonious probabilistic classifier able to prospectively identify the C1–C5 DLBCL subtypes in newly diagnosed patients [67].

### 2.3. Tumor Microenvironment and Angiogenesis

Based on several pieces of compelling evidence highlighting the impact of the DLBCL niche in nursing cancer cells, by promoting a favorable stromal environment, several prospective clinical studies are needed to validate the clinical utility of the stromal gene expression profile in DLBCL and dissect subtypes which would profit the most from anti-angiogenic and milieu-targeting strategies [68,69]. Nonetheless, it is well known that the presence of immune and inflammatory cells contributes to modulate tumor growth and invasion in hematological malignancies and DLBCL [70,71,72]. Analysis of the tumor microenvironment is an important aspect in the assessment of progression of DLBCL. Different components of the microenvironment are considered in DLBCL including mast cells and TAMs to establish several correlations among prognostic significance, stage-related tumor progression, and differences in treatment outcome [73,74].

Lymphomas include more than 40 lymphoproliferative disorders, and angiogenesis plays a critical role in their progression and prognosis [75,76].

The state-of-the-art knowledge of the crucial mechanisms promoting angiogenesis and mediating immunosuppression during DLBCL development, progression [77,78], and sensitivity to drugs [26,79] needs further in-depth analysis. Solid and hematological neoplasms propagate and progress through several vicious cycles, feeding into the surrounding tumoral milieu [80,81,82,83], and emergent knowledge pinpoints angiogenesis and immunosuppression as simultaneous processes in response to this reciprocal loop [84,85] and to a plethora of paracrine and exogenous stimuli [86,87,88]. Lymphoproliferative disorders [89,90] and DLBCL [91] are no exception. Accordingly, strategies combining anti-angiogenic therapy and immunotherapy seem to have the potential to tip the balance of the tumor microenvironment and improve the treatment response of lymphoid malignancies [21,22,92,93]. These pieces of evidence prompted an intense translational investigation aimed at targeting angiogenesis and the immune system in a coordinated fashion, based on the preclinical insights available [94,95].

### 2.4. Increased Vascularization, VEGF Expression and MicroRNA (miRNA)

The presence of an increased number of immature vessels in DLBCL compared with follicular lymphoma (FL) was demonstrated [96]. ABC DLBCL CD5+ showed higher microvascular density (MVD) than GCB DLBCL [97]. MVD was higher in CD5+ DLBCL in comparison with the CD5− subgroup [98].

Transformation from indolent B-cell lymphoma to aggressive DLBCL and poor prognostic subgroups within DLBCL is associated with increased VEGF expression [99]. In aggressive subtypes of DLBCL, VEGF-A-producing CD68+ VEGFR1+ myelo-monocytic cells are closely associated with newly formed blood vessels [68]. In DLBCL, the average MVD correlates with the intensity of VEGF, VEGFR-1, and VEGFR-2 expression in tumor cells [100]. Other studies in DLBCL found no correlation between MVD and VEGF expression [101]. The transcript level of the soluble isoforms of VEGF, such as VEGF121, has a major impact on the prognosis of ABC-like DLBCL, whereas low VEGF121 expression was associated with a significantly better survival than high expression [91]. Moreover, 57 genes involved in immune response and T-cell activation were decreased in patients with high VEGF121 expression in both ABC-like and GBC-like subtypes of DLBCL [91].

In a meta-analysis of eight studies conducted on 670 patients, positive VEGF expression in blood-circulating lymphocytes and lymph nodes correlated with shorter survival in newly diagnosed DLBCL [102]. In another study performed on 149 newly diagnosed DLBCLs, high serum VEGF level was associated with poorer prognosis [103]. VEGF-A- and VEGFR-1-negative patients had an improved overall survival compared to VEGF-A- and VEGFR-1-positive ones [104]. Polymorphism in the VEGFR-2 gene may be associated with better survival in DLBCL patients [105].

Borges et al. [106] demonstrated an association between increased expression of pro-angio miRs miR-126 and miR130a, along with anti-angio miR-328, and the subtype non-GCB. Moreover, they found higher levels of the anti-angio miR-16, miR-221, and miR-328 in patients with low MVD and a stromal 1 signature.

More recently, Lupino et al. [107] demonstrated that the overexpression of SPHK1, one of the two isozymes responsible for the production of sphingosine-1 phosphate (SP1), a bioactive sphingolipid metabolite acting as a potent inducer of angiogenesis [108], correlates with an angiogenic transcriptional program in DLBCL.

### 2.5. Correlations among Angiogenesis, VEGF Expression, and Response to Therapy

Immunodeficient mice engrafted with human DLBCL treated with antibodies against human or murine VEGFR-1 or VEGFR-2 showed a significant 50% reduction in tumor mass after treatment with human anti-VEGFR-1. By contrast, inhibition of murine VEGFR-1 resulted in a similar tumor reduction, but inhibition of human VEGFR-2 had no antitumor effect [109].

In patients affected by DLBCL treated with anthracycline-based chemotherapy, no correlation between increased MVD and VEGF expression in tumor cells was demonstrated. Moreover, high VEGF and VEGFR-1 expression identified a subgroup of patients affected by DLBCL with improved overall survival and progression-free survival [100]. In patients with DLBCL treated with R-CHOP, a high serum level of VEGF was associated with adverse outcome, having lower values in survivors than in non-survivors [110]. Additionally, high MVD determines a poor outcome in DLBCL in patients treated with R-CHOP [97]. Bevacizumab inhibits tumor growth, either alone or in combination with chemotherapy, in untreated DLBCL [111].

### 2.6. Targeting Angiogenesis and the Immune System in DLBCL: A Single-Center Experience

**Figure 1:**
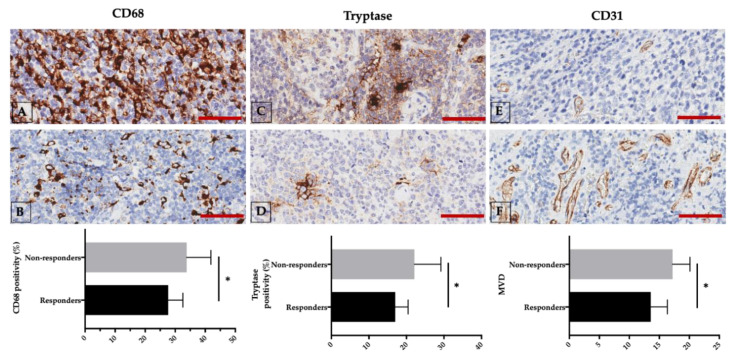
Non-responder (upper panels (**A**,**C**,**E**) and responder newly diagnosed diffuse large B-cell lymphoma (DLBCL) patients (middle panels (**B**,**D**,**F**) are characterized by different CD68, tryptase, and CD31 expression. Lower panels: respective comparison of non-responder and responder groups. Scale bar: 50 μm. * *p* < 0.05, assessed by Mann–Whitney test. Representative images from 29 untreated DLBCL patients are presented [112].

Recently, we demonstrated that there is a significant increase in tryptase-positive mast cells and CD68-positive TAMs, as well as a significant increase in MVD and a positive correlation in chemo-resistant non-responder when compared with chemo-sensitive responder DLBCL patients (Figure 1) [112].

**Figure 2:**
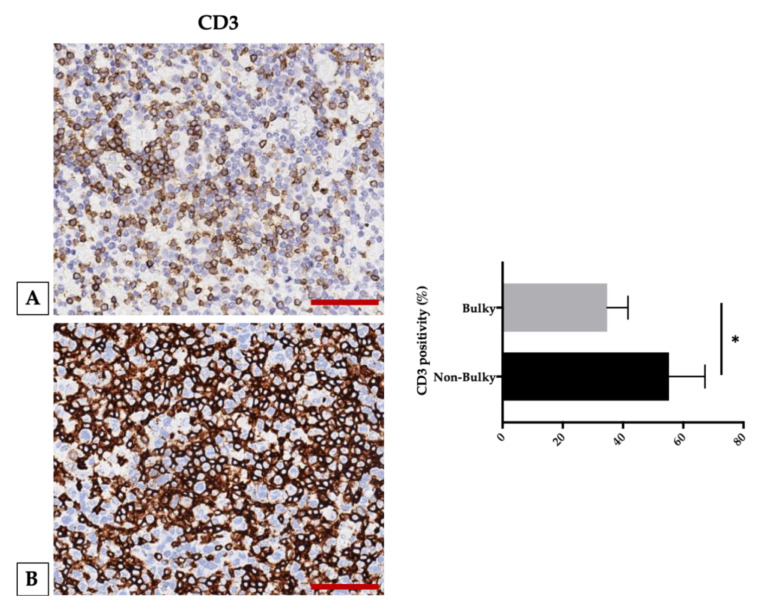
CD3 expression in bulky and non-bulky DLBCL. Left panel: (**A**) Representative image of CD3 expression in a case with bulky involvement. (**B**) Representative image of CD3 expression in a case with non-bulky DLBCL. Right panel: comparison between bulky and non-bulky disease groups with a significant difference between the groups in the CD3 infiltrate. Scale bar: 50 μm. * *p* < 0.05, assessed by Mann–Whitney test. Representative images from 29 untreated DLBCL patients are presented [113].

Moreover, we uncovered CD3-positive T cells to be decreased while comparing bulky (patients with bulky disease are defined by the presence of a large nodal tumor mass >10 cm or mediastinal disease) and non-bulky groups (Figure 2) [113], suggesting that a reduction in T cells in bulky disease patients contributes to loosen the immune control over the tumor, resulting in increased cell proliferation and large tumor masses [114].

**Figure 3:**
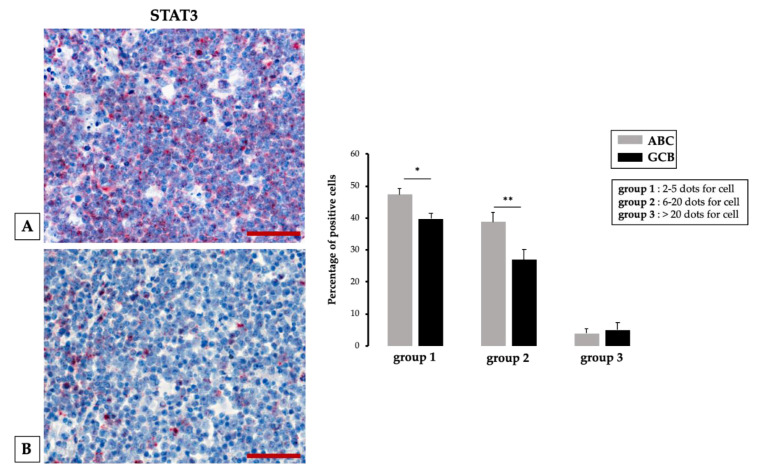
Left panel: different STAT3 expression in histological samples from activated B cell (ABC) (**A**) and germinal center B cell (GCB) (**B**) DLBCL assessed by RNAscope. Scale bar: 60 μm. Right panel: quantification of RNA ISH staining of STAT3 messenger RNA (mRNA) positivity in ABC and GCB DLBCL samples. The percentage of STAT3 mRNA expression significantly increases in the ABC group 1 and 2 tumor samples compared to GCB; * *p* < 0.05; ** *p* < 0.01, assessed by Mann–Whitney test. Representative images from 30 untreated DLBCL patients are presented [115].

Likewise, we demonstrated, comparing by means of RNA scope technology, STAT3 RNA expression in two selected groups of ABC DLBCL and GBC DLCBCL, that ABC tissue samples contained a significantly higher number of STAT3-positive cells than GBC tissue samples (Figure 3) [115].

**Figure 4:**
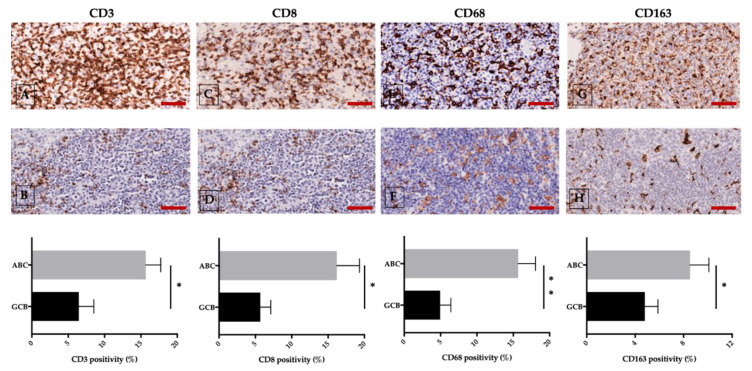
ABC (upper panel) and GCB (middle panel) DLBCL different expression of CD3 (**A**,**B**), CD8 (**C**,**D**) CD68 (**E**,**F**), and CD163 (**G**,**H**) assessed by immunohistochemical staining. The morphometric analysis is expressed as marker percentage positivity (lower panel). Scale bar: A–H 60 μm. Representative images from 60 untreated DLBCL patients are presented; * *p* < 0.05; ** *p* < 0.01, assessed by Mann–Whitney test [116].

Furthermore, through microscopic imaging, we uncovered tumor vessels in ABC samples but not GBC samples to be coated by FVIII- and STAT3-positive endothelial cells [115]. Evidence from our group revealed a positive correlation not only between STAT3 expression and CD3, CD8, and CD68, but also between D163-positive cells in the ABC and the GBC groups (Figure 4) [116].

**Figure 5:**
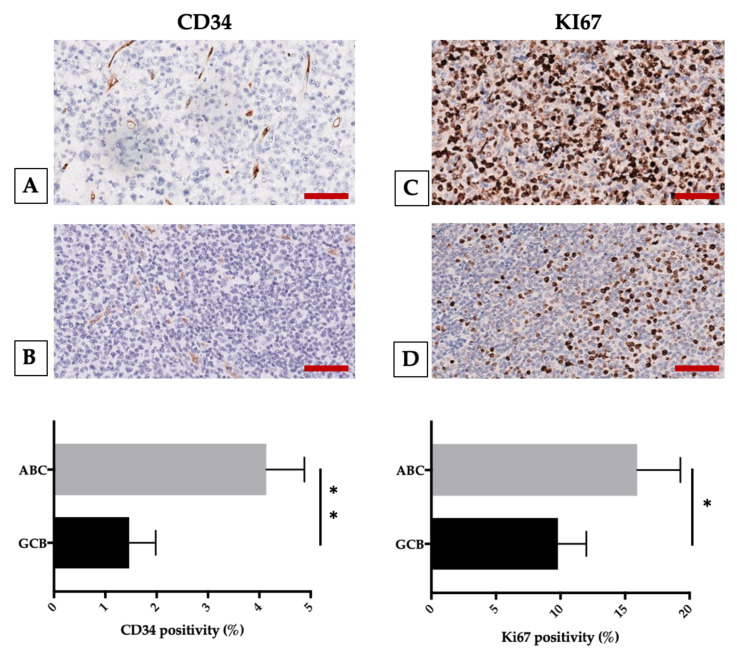
ABC (upper panel) and GCB (middle panel) DLBCL different expression of CD34 (**A**,**B**) and Ki67 (**C**,**D**) assessed by immunohistochemical staining. The morphometric analysis is expressed as marker percentage positivity (lower panel). Scale bar: A–D 60 μm. Representative images from 60 untreated DLBCL patients are presented; * *p* < 0.05; ** *p* < 0.01, assessed by Mann–Whitney test [116].

Additionally, in the ABC group, we found also a positive correlation between CD8- and CD34- and between Ki67- and CD68/CD163-positive cells (Figure 5).

## 3. Discussion

Overall, data generated by our group corroborated previous findings, pointing toward a higher STAT3 expression being associated with higher CD163- and CD8-positive cell infiltration, which induces a strong angiogenic response in ABC DLBCL as compared with GCB DLBCL [116]. Preliminary results generated in our and other labs uncovered enhanced angiogenesis to be a strong regulator of lymphoproliferative disorder prognosis due to direct and indirect activation of cell survival [115,116,117]. The cell-adhesion-dependent DLBCL milieu interaction nurses DLBCL proliferation, by supporting immune-surveillance evasion [118]. Independent data provided compelling evidence that, in the intimate interaction between stromal cells, the malignant clone creates a permissive immune microenvironment within the lymphoma niche, which starts a vicious cycle hijacking anti-tumor activity [21,119,120]. Mechanistically, endothelial cells, by expressing TIM-3, HB-EGF [120,121,122], and a plethora of surface and soluble factors, prompt defective immunosurveillance and, in turn, allow for the persistence and proliferation of lymphoid neoplastic cells [123,124,125], envisioning novel therapeutic windows [126,127]. Moreover, the initial observation that the expression level of the adhesion molecules by the malignant lymphoma cells can predict disease outcome in extranodal DLBCL [128] prompted further investigation, especially in peculiar clinical disease phenotypes, such as DLBCLs involving the central nervous system (CNS) [128]. Remarkably, CNS spreading represents a paradigmatic extranodal localization with peculiar pathobiology involving adhesion molecule deregulated expression [129] and hyperactivation of the angiogenesis fueling pathway [130] along with a truncal genomic signature [131], which can contribute to drug sensitivity and resistance [132,133,134], as in other malignancies [135,136,137]. Therefore, given that the aberrant expression of adhesion molecules on bone marrow endothelial cells of patients with lymphoid and myeloid neoplasia was also discovered to predict poor clinical outcome [138,139,140,141], it is tempting to speculate a vicious cycle in DLBCL by paralleling the neoplastic cell behavior [128], whereby the described molecular signature [36,142] has more interactions among themselves than what would be expected for a random set of gene-encoding proteins drawn from the genome [143].

Based on these findings and on several pieces of compelling evidence investigating how the deregulated adhesion-mediated system would contribute to more aggressive disease, several attempts uncovered the junctional adhesion molecule role in mediating disease aggressiveness [141,144,145]. In line with previous results [128,146,147], preliminary data from our lab demonstrate that direct contact of environmental cells with DLBCL cells would enhance adhesion molecule levels, thus preventing both direct and indirect cell invasiveness and epithelial–mesenchymal transition and extra-nodal dissemination (unpublished data). Even more interesting, the cell adhesion molecule junctional adhesion molecule-A (JAM-A) presents remarkable features [148], whereby it can interact with itself if expressed on two opposing cell types. Furthermore, if JAM-A is shed by a cell, the soluble form of the JAM-A molecule can bind to cell-bound JAM-A, which in turn notably enhances its binding capacity [149,150,151]. Remarkably, consistently with Peng-Peng Xu et al. [128], JAM-A appears related to extra-nodal involvement in DLBCL, being selectively expressed in those cases. The therapeutic effects of blocking angiogenesis, the endothelial adhesion system, JAM-A, and its cognate shedding regulator ADAM17 were mainly observed in preclinical models but not in patients and, therefore, they must be interpreted with caution [151,152,153,154,155]. In a clinical setting, the adhesion system and neoangiogenesis, along with competent CD8 T cells and dendritic cells, had increased OS and time to progression [99]. Thus, it is likely that invasiveness potential, along with new blood vessel formation (i.e., angiogenesis) within the DLBCL environment, is a recognized hallmark of disease progression, mirroring cancer evasion from T-cell immune surveillance [156]. Endothelial progenitor cell trafficking was uncovered to be implicated in DLBCL progression [157,158], especially in early disease phases [100,159]. Several clinical trials in DLBCL tested the effects of angiogenesis-targeting agents, such as bevacizumab, which are used in combination with other agents, including B-cell-targeting agents [101,160,161]. Nonetheless, the lack of clinical effect in the randomized study gained by the addition of an anti-angiogenic approach to chemo-immunotherapy involving the tumor milieu might be predictive of the response to anti-angiogenesis in DLBCL, being beneficial in DLBCL with a high relative expression of a set of endothelial markers and angiogenic gatekeepers (the “stromal 2” subtype), correlating with enhanced vasculogenesis [17,69]. Furthermore, since compelling evidence pinpoints structural abnormalities in the endothelium as impairing antitumor immunity by forming barriers to immune surveillance [162], the tumor-associated endothelium is currently also described as a caretaker that synchronizes the entrance and egress of the immune cells within the neoplastic niche [163,164]. Therefore, while defining DLBCL also based on the quantity and quality of immune cell infiltrates might provide novel rationale to overcome the lack of clinical success gained by angiogenesis-targeting agents so far, identifying the abnormalities in the DLBCL endothelium impairing the crosstalk with adaptive immunity may also be valuable. Targeting these abnormalities can improve the success of immune-based therapies for different cancers, as well as DLBCL, by improving immune–vascular crosstalk for DLBCL, enhancing anti-lymphoma immunotherapy using anti-angiogenesis [165]. Thus, further studies of anti-angiogenic approaches in B-NHL and DLBCL should not be denied [161]. Indeed, while preventing secondary immunodeficiencies [166,167], this evidence provides the translational rationale to overcome the scanty effect of the anti-angiogenic approach in DLBCL obtained so far by novel angiogenesis targeting via RAS pathway inhibition, while combining immune-modulatory agents (IMiDs, i.e., lenalidomide) when appropriate [168,169,170]. Assuming the different angiogenic impacts on a given disease stage, it would be worth tailoring the vasculogenic manipulation in early DLBCL with the high-risk phenotype [78]. In this frame of thinking, one critical effect of corrupted angiogenesis is represented by disease dissemination, within and outside the original niche localization, driving intra- and extra-nodal adhesion-dependent manifestation in DLBCL. Finally, the judicious use of anti-angiogenics to normalize tumor vasculature might represent a strategy reprograming the tumor microenvironment to improve next-generation immunotherapy for DLBCL.

## 4. Conclusions

Lymphomas constitute a large group of more than 40 lymphoproliferative disorders, classified on the basis of morphologic, immunologic, genetic, and clinical criteria. The importance of the tumor milieu and angiogenesis in lymphoproliferative disorders was studied in relation to their impact on the prognosis of patients, suggesting high relevance in different types of lymphomas. Literature data concerning the angiogenesis of NHL are limited compared with HL, with most studies performed by retrospective immunohistochemical analysis, where evidence of correlation between cellular components of the microenvironment and increased vascularity was established. Within the different types of B-cell lymphomas, angiogenesis may be prominent in aggressive rather than indolent subtypes.

Current frontline DLBCL therapy although fairly successful (70–80% remission rates with the standard R-CHOP chemotherapy regimen) is frequently followed by relapse (40% of cases within 2–3 years), with an often refractory DLBCL. Anti-angiogenic therapy and microenvironment-directed therapy represent important tools for the treatment of human lymphomas. However, a significant number of patients are resistant, whereas those who respond have minimal benefits. Nevertheless, these new findings may point toward a potential Achilles heel of DLBCL which, in the future, might be exploited therapeutically in the relapsed/refractory setting and in extranodal dissemination.

## Abbreviations

ADAM17	Disintegrin and metalloproteinase domain-containing protein 17
BCL2	B-Cell CLL/Lymphoma 2
BCL6	B-Cell Lymphoma 6 Protein
BCR	B-Cell Receptor
BRD	Bromodomain
CARD11	Caspase Recruitment Domain Family Member 11
CD68	cluster of differentiation 68
CN	Copy number
CREB	cAMP-response element-binding protein
CREBBP	CREB Binding Protein
DDX3X	DEAD-Box Helicase 3 X-Linked
DEAD	DEAD-box helicase family
EZH2	Enhancer Of Zeste Homolog 2
FISH	Fluorescence in situ hybridization
FVIII	Factor VIII
GNA13	G Protein Subunit Alpha 13
HAT	Histone Acetyltransferase
HB-EGF	Heparin-binding EGF-like growth factor
Ig	Immunoglobulin
IRF4	interferon regulatory factors 4
ISH	In situ hybridization
KMT2D	Lysine Methyltransferase 2D
KO	Knock out
LMO2	LIM domain only 2 (rhombotin-like 1)
m3D-IPI)	three-risk group model International Prognostic Index
MIB1	Mindbomb E3 Ubiquitin Protein Ligase 1
MUM1	melanoma associated antigen (mutated) 1
MYC	Myc-Related Translation/Localization Regulatory Factor
MYD	Myeloid Differentiation Primary Response Gene
MYD88	Myeloid Differentiation Primary Response Gene (88)
NF-κB	Nuclear Factor Kappa B Subunit
NGS	next generation sequensing
NOS	nitric oxide synthase
NOTCH2	Neurogenic Locus Notch Homolog Protein 2
OS	Overall Survival
PFS	Progressiob free Survival
PTEN	Phosphatase And Tensin Homolog
RAS	Rat Sarcoma Viral Oncogene Homolog
SPEN	SPEN family transcriptional repressor (
SPHK1	Sphingosine Kinase 1
STAT3	Signal Transducer And Activator Of Transcription 3
SUZ12	Suppressor Of Zeste 12 Protein Homolog
TIM-3	T-cell immunoglobulin mucin-3
TNFAIP3	Tumor necrosis factor, alpha-induced protein 3
TP53	Tumor Protein P53
